# HIV-related knowledge, perceptions, attitudes, and utilisation of HIV counselling and testing: a venue-based intercept commuter population survey in the inner city of Johannesburg, South Africa

**DOI:** 10.3402/gha.v8.26950

**Published:** 2015-04-28

**Authors:** Lucy Chimoyi, Ndumiso Tshuma, Keith Muloongo, Geoffrey Setswe, Bismark Sarfo, Peter S. Nyasulu

**Affiliations:** 1 Wits Reproductive Health and HIV Research Institute (Wits RHI), Faculty of Health Sciences, School of Clinical Medicine, University of the Witwatersrand, Johannesburg, South Africa; 2 Community AIDS Response, Norwood, Johannesburg, South Africa; 3 HIV/AIDS, STI and TB (HAST) Research Program, Human Sciences Research Council (HSRC), Pretoria, South Africa; 4 Department of Health Studies, University of South Africa (UNISA), Pretoria, South Africa; 5 Department of Epidemiology and Disease Control, School of Public Health, College of Health Sciences, University of Ghana, Legon-Accra, Ghana; 6 School of Health Sciences, Monash University, Ruimsig, Johannesburg, South Africa; 7 Faculty of Health Sciences, School of Public Health, University of the Witwatersrand, Johannesburg, South Africa

**Keywords:** HCT, Johannesburg, commuter population, HIV/AIDS, stigma

## Abstract

**Background:**

HIV counselling and testing (HCT) and knowledge about HIV have been key strategies utilised in the prevention and control of HIV/AIDS worldwide. HIV knowledge and uptake of HCT services in sub-Saharan Africa are still low. This study was conducted to determine factors associated with HCT and HIV/AIDS knowledge levels among a commuter population in Johannesburg, South Africa.

**Objective:**

To identify the factors associated with HCT uptake among the commuter population.

**Design:**

A simple random sampling method was used to select participants in a venue-based intercept survey at a taxi rank in the Johannesburg Central Business District. Data were collected using an electronic questionnaire. Logistic regression analysis assessed factors associated with HIV testing stratified by gender.

**Results:**

1,146 respondents were interviewed, the maority (n=579, 50.5%) were females and (n=780, 68.1%) were over 25 years of age. Overall HCT knowledge was high (*n*=951, 83%) with more females utilising HCT facilities. There was a significant difference in HIV testing for respondents living closer to and further away from health facilities. Slightly more than half of the respondents indicated stigma as one of the barriers for testing (*n*=594, 52%, *p*-value=0.001). For males, living with a partner (aOR: 1.68, 95% CI: 1.02–2.78, *p*-value: 0.041) and possessing a post-primary education were positively associated with testing (aOR: 2.00, 95% CI: 1.15–3.47, *p*-value: 0.014), whereas stigma and discrimination reduced the likelihood of testing (aOR: 0.40, 95% CI: 0.31–0.62, *p*-value: <0.001). For females, having one sexual partner (aOR: 2.65, 95% CI: 1.19–5.90, *p*-value: 0.017) and a low perceived benefit for HIV testing (aOR: 0.54, 95% CI: 0.30–0.96, *p*-value: 0.035) were associated with HIV testing.

**Conclusion:**

The overall HIV/AIDS knowledge was generally high. Gender-specific health education and HIV intervention programmes are needed for improved access to HCT services. One favourable intervention would be the use of home-based HCT programmes.

Approximately, 35 million people are currently living with HIV globally (1). Although there is a general decline of HIV infections worldwide, sub-Saharan Africa (SSA) accounts for about 70% of all new HIV cases reported in 2012 (1). South Africa reportedly has the largest burden of HIV/AIDS in the world (2) and has embarked on a number of approaches aimed at reducing this burden, such as increasing HIV-related knowledge which could lead to the reduction of risky behaviours. HIV counselling and testing (HCT) has been one of the strategies identified as necessary for HIV intervention across SSA which also provides a gateway for treatment of people living with HIV/AIDS (PLWHA) (3, 4). HCT provides an avenue for education and promotion of behaviour change, resulting in a reduction in risk behaviours (5–7). Novel approaches to HCT such as routine HCT, home-based HCT (8, 9), provider-initiated HCT (10, 11), client-initiated HCT (12), use of community-based lay counselling (13), and couples counselling and testing (14) have been added to the traditional facility-based HCT delivery systems (2–4, 6, 7, 10, 12, 15–20). Despite this variety of delivery approaches to HIV intervention and the apparent advantages associated with HCT, the uptake of HIV testing as well as counselling remains relatively low in SSA, especially among males (2–4, 6, 7).

The major barriers to HCT use among men were poor utilisation of voluntary HIV counselling and testing (VCT) services due to poor access, stigma, and confidentiality of services (15). Research conducted in both developing and developed countries has identified social and economic barriers as well as health system factors as impediments to accessing HCT services (2, 4). Several possible contributing factors known to influence uptake of HCT are socio-demographic characteristics (21), nearness to a health facility, HIV-related awareness and knowledge, perception of being at risk of HIV infection, perceived benefits of HCT, and psychosocial factors such as HIV/AIDS-related stigma and discrimination and anxieties about confidentiality (2, 21, 22).

The majority of the people living in South Africa are aware of HCT (2, 22, 23), but many of them shun HIV testing because of the negative perceptions associated with testing services. Health system factors have been identified as barriers to HIV testing (15). A study conducted in Uganda identified a lack of confidentiality due to health workers’ apathy and the quality of HIV results as important factors which reduced VCT utilisation (15).

AIDS-related stigma and discrimination, factors that influence decisions surrounding the uptake of HCT and HIV treatment services (24–26), have profound effects on an individual by reducing his or her willingness to engage in not only HIV testing but also treatment and prevention that would improve such an individual's quality of life (22). Various studies have acknowledged the need to educate communities on the benefits of testing to reduce stigma associated with HIV testing and being HIV-positive (27–29). A survey conducted in Nigeria, which assessed the HIV-related stigma among two main ethnic groups, revealed that female participants were less likely to utilise HCT for fear of stigmatisation from partners (30). A cluster randomised controlled trial conducted in urban and rural South Africa suggested a link between HIV testing and stigma in which low levels of stigma increased the uptake of HIV testing (22). Since these studies have shown a strong association between stigma and testing for HIV, HIV infections can be reduced by identifying and controlling factors which play critical roles in this association (31).

Further studies have shown that people who perceived their HIV risk as low were less likely to take up HCT services (3, 31). A study in a South African urban township showed that individuals who were aware of antiretroviral therapy (ART) were more likely to utilise HCT service (23). However, other studies indicated that, despite the introduction of free ART, the use of HCT services remains persistently low in many African countries (32).

Several factors militating against HCT uptake services by adult populations have been identified. However, little can be said about the commuter population uptake of HCT services, even though they form an important group not to be ignored in the fight against HIV transmission. Administration of health services to this group of people is challenging as they leave their homes early in the morning and return late at night. There is need to identify factors that will help in scaling up the use of HCT services among this group of individuals. Against this background, the objective of this study was to determine the level of HIV/AIDS knowledge, attitudes, and perceptions and also to explore factors associated with the use of HCT services among a commuter population in the inner city of Johannesburg in South Africa.

## Methods

### Study design

A venue-based intercept survey was conducted at the four corners of the Noord Street taxi rank in Johannesburg's inner-city Central Business District (CBD). A simple random technique to select study participants was used which involved selecting an even number from a closed bowl containing folded paper numbered 1 to 10. A total of eight trained field workers were positioned at the four corners of the taxi rank that served as intercept points. The field workers approached individuals as they passed through the intercept points between 09:00 and 16:00 on each of the 2 survey days. Verbal consent was sought from potential participants before picking a number from a bowl. Individuals who selected even numbers were allowed to respond to a closed-ended questionnaire in plain English.

### Study setting

The taxi rank is Johannesburg's primary transit centre for all passenger service vehicles operating in South Africa's mini-bus system. The Noord Street taxi rank, which is situated at the heart of the inner city, is the largest due to the influx of people from different areas. It is used as a gateway to different areas of the city. The inner city has been transformed from a spacious, well-maintained business district to a densely populated, culturally diverse, and poorly regulated city centre. This situation has been propagated by the migration of individuals from not only other parts of South Africa but also other countries in search of economic opportunities in Johannesburg. It is well known that in as much as taking a mini-bus is often inconvenient and time consuming, it is a more cost-effective way of getting around the city. As such, the mini-bus system is mostly used by individuals without private cars or those who cannot afford the more expensive option of using ‘metered taxis’. Although a high number of individuals commute through this taxi rank, a limited number of health intervention studies have been conducted on the commuter population.

### Data collection

Data were collected using an electronic questionnaire loaded in Tablet personal computers over a 2-day HCT community outreach campaign that targeted taxi ranks in the Johannesburg Metropolitan District. Trained fieldworkers interviewed participants in May 2013 and recorded responses directly into Tablets.

### Measures

All measures in this study were self-reported. The dependent variable, uptake of HCT, was measured by asking whether the respondent ever tested for HIV (0=‘No’ and 1=‘Yes’). The independent variables included (1) socio-demographic characteristics: gender (male, female), age group (≤25 years, >25 years), marital status (single, cohabiting/married, divorced), employment status (unemployed, employed), education level (primary, secondary, tertiary), sexual partnerships in the last 3 months (none, one, more than one), distance to nearest clinic (<20 km, >20 km), and affected by HIV by having knowledge of a family member or friend with HIV infection (no, yes). (2) Factors related to knowledge and perceptions: knowledge of HCT (no, yes), perceived benefits for HIV testing (low, high), and perceived risk for HIV infection (low, high). Reasons for low perception of HIV risk included use of condoms and/or birth control pills, faithfulness, and recent testing. There were stigma and discrimination responses to the question regarding the social barriers related to HIV testing. (3) Health service factors: lack of confidentiality at testing stations (nearness of testing station to the home or workplace, fear of discrimination, and lack of confidentiality at testing stations). Health workers who reduced the willingness to test were measured as nurses, doctors, matrons, or counsellors; and lack of ART services was measured by selecting ‘lack of information on ART’ or ‘low or no supply of ART’.

### Data analysis

All analyses were conducted using Stata version 12.0 (33). The analysis examined the relationship between the outcome of interest (tested for HIV) and the independent variables of socio-demographic characteristics, HIV-related knowledge, perceptions, attitudes, and utilisation of HCT services. The analyses were conducted using a three-pronged approach. (1) Descriptive statistics of history of HIV testing were calculated with respect to measures of socio-demographic characteristics, assessment of HIV-related knowledge, perceptions, attitudes, and health services factors. In addition, chi-square (χ^2^) was used to explore the factors associated with having taken an HIV test and the assessment of HIV-related knowledge, perceptions, and attitudes. (2) To assess the relative contribution of each of these predictor variables, a logistic regression analysis was carried out. Univariate logistic regression analysis examined the association between these variables and the history of HIV testing. This allowed for understanding the effect of each independent variable without controlling for confounding variables. A reference level was selected for categorical variables with multiple responses in which other levels of the variable were subsequently compared. (3) Socio-demographic, knowledge assessment, and health factor variables that were significant (*p*<0.1) were entered into a model for multiple logistic regression analysis using the backward elimination method to control for confounding. A stepwise model selection procedure was adopted to determine the factors associated with HCT uptake in the sample population. To accommodate the different factors unique to each gender, separate logistic regression models were fitted for male and female participants. Associations between the outcome and independent variables were assessed using odds ratios, 95% confidence limits, and *p*-values and were presented by gender. A *p*-value ≤0.05 was deemed statistically significant. Model fit was assessed using the Hosmer–Lemeshow statistic, with a *p*-value>5% denoting a good fit.

## Results

### Participant characteristics

A total of 1,146 participants were interviewed during this survey (Table 1). The sample consisted of (*n*=579, 50.5%) women and (*n*=567, 49.5%) men, and a majority of the women (53.8%) had tested for HIV compared with only 46.2% of men. The majority of the survey participants were older than 25 years of age (*n*=780, 68.1%), single (*n*=801, 69.9%), and in monogamous relationships (*n*=681, 59.4%). In addition, most of the participants were employed (*n*=672, 58.6%), possessed a secondary school education (*n*=498, 43.5%), had been affected by HIV (*n*=882, 77.0%), and lived within 20 km of their nearest clinic (*n*=972, 84.8%).

**Table 1:** Socio-demographic characteristics and univariate associations of uptake of HCT among survey participants (*N*=1,146)

Factor	Total (*N*)	Not HIV tested, *n* (%)	HIV tested, *n* (%)	*P*-value*	Crude OR	95% CI	*P*-value
Gender							
Female	579	81 (36.82)	498 (53.78)	<0.001*	1 (Ref)		
Male	567	139 (63.18)	428 (46.22)		0.50	0.37–0.68	<0.001**
Age group							
Less than 25	366	87 (39.55)	279 (30.13)	0.007*	1 (Ref)		
More than 25	780	133 (60.45)	647 (69.87)		1.56	1.12–2.06	0.007**
Marital status							
Single	801	166 (75.45)	635 (68.57)	0.065	1 (Ref)		
Cohabiting/married	321	48 (21.82)	273 (29.48)		1.49	1.05–2.11	0.027**
Divorced	24	6 (2.73)	18 (1.94)		0.78	0.31–2.01	0.612
Employment status							
Unemployed	474	96 (43.64)	378 (40.82)	0.446	1 (Ref)		
Employed	672	124 (56.35)	548 (59.18)		1.12	0.83–1.51	0.446
Education level							
Primary	324	85 (38.64)	239 (25.81)	0.001*	1 (Ref)		
Secondary	498	81 (36.82)	417 (45.03)		1.83	1.30–2.58	0.001**
Tertiary	324	54 (24.54)	27 (29.16)		1.78	1.21–2.61	0.003**
Sexual partnerships							
None	108	33 (15.00)	75 (8.10)	<0.001*	1 (Ref)		
One	681	102 (46.36)	579 (62.53)		2.50	1.58–3.96	<0.001**
More than one	357	85 (38.64)	272 (29.37)		1.41	0.87–2.27	0.159
Affected by HIV							
No	264	58 (26.36)	206 (22.25)	0.192	1 (Ref)		
Yes	882	162 (73.64)	720 (77.75)		1.25	0.89–1.75	0.193
Distance to nearest clinic							
Less than 20 km	972	162 (73.64)	810 (87.47)	<0.001*	1 (Ref)	0.28–0.58	<0.001**
More than 20 km	174	58 (26.36)	116 (12.53)		0.40		

* Bivariate associations determined by chi-square tests at 5% significance level.

** Variables with *P*-value of equal to or less than 5% significance level were entered into multiple logistic regression models.

### Assessment of HIV-related knowledge, attitudes, perceptions, and utilisation of HCT

The distribution of respondents regarding HIV-related knowledge, perceptions, attitudes, and utilisation of HCT services was assessed. The response with the highest frequency was included in Table 2. The majority (*n*=951, 83%) of the participants were aware of the HCT and PICT services. Of these, (*n*=812, 85%) had tested for HIV. Slightly more than half of the respondents perceived the risk of HIV infection as low (*n*=594, 52%), and the majority responded that using condoms was their preferred method for HIV prevention (*n*=876, 76%). There was a high perception for HIV testing reported (*n*=738, 64.4%). Slightly more than half of the survey participants (*n*=594, 52%) cited stigma or negative attitudes as a factor that would deter HIV testing, and ignorance (*n*=531, 46%) was the main reason why a lot of HIV-related stigma and discrimination prevailed in this study population. Most of the survey respondents (*n*=1,023, 89%) reported that the lack of information on ART services at clinics and long queues at health centres (*n*=711, 62%) affected accessibility of HCT services. Slightly more than half of the respondents indicated that living close to the HCT centres contributed to the lack of confidentiality at testing stations (*n*=615, 54%). Nurses (*n*=660, 58%) were cited as the main reason for unwillingness to test for HIV. Few respondents had paid a fee for HIV testing (*n*=141, 12%).

**Table 2:** HIV-related knowledge and perceptions, stigma, and health factors affecting the uptake of HCT among study participants

Variable	Overall, *N* (%)	Not HIV tested, *n* (%)	HIV tested, *n* (%)	*P*-value	Crude OR	95% CI	*P*-value
Knowledge, stigma, and perceptions							
Are you aware of HCT, and have you heard of PICT? (yes)	951 (82.98)	139 (14.62)	812 (85.38)	<0.001*			
What is your perception of the risk of getting HIV infection? (low)	594 (51.83)	123 (16.67)	471 (83.33)	0.178	–	–	–
What makes you think you can't be HIV-positive? (I use condoms)	876 (76.44)	166 (18.95)	710 (81.65)	<0.001*	–	–	–
What is your perceived benefit of doing an HIV test? (high)	738 (64.40)	123 (16.67)	615 (83.33)	0.003*	0.74	0.52–1.05	0.091**
What are your perceptions for HIV testing? (a lot of stigma associated with HIV testing)	594 (51.83)	97 (16.33)	497 (83.67)	<0.001*			
What are the social barriers to HIV testing? (stigma and discrimination)	429 (37.43)	112 (26.11)	317 (73.89)	<0.001*	0.50	0.37–0.68	<0.001**
Why is there stigma and discrimination related to HIV testing? (ignorance)	531 (46.34)	111 (20.90)	420 (79.10)	0.163			
Health factors							
Why would you say there is a lack of ART services? (no information on ART services)	1,023 (89.27)	201 (19.65)	822 (80.35)	0.264			
Why do you think there is no confidentiality at testing stations? (testing station located near place of residence)	615 (53.66)	102 (16.59)	513 (83.41)	<0.001*			
Which health worker reduces your willingness to test? (nurses)	660 (57.59)	135 (20.45)	525 (79.55)	0.055			
Experience difficulty in accessing HCT services? (yes)	1,023 (89.27)	196 (19.16)	827 (80.84)	0.925			
Have you paid for HIV testing? (yes)	141 (12.30)	21 (14.89)	120 (85.11)	0.166	1.41	0.87–2.30	0.168

* Bivariate associations determined by chi-square tests at 5% significance level.

** Variables with *P*-value of equal to or less than 5% significance level were entered into multiple logistic regression models.

### Factors associated with utilisation of HCT services among the study population

The results of univariate and multivariate logistic regression of factors associated with HIV testing as the outcome variable were presented in Tables 1–3. Univariate analyses revealed that men, compared to women, were less likely to be tested for HIV (OR=0.50; 95% CI: 0.37–0.68) and that living more than 20 km from the nearest clinic reduced the chance for HIV testing (OR=0.40; 95% CI: 0.28–0.58). Respondents who were older than 25 years (OR=1.56; 95% CI: 1.12–2.06), were married or cohabiting (OR=1.49; 95% CI: 1.05–2.11), possessed a post-primary education (OR=1.83; 95% CI: 1.30–2.58 and OR=1.78; 95% CI: 1.21–2.61), and had one sexual partner (OR=2.50; 95% CI: 1.58–3.96) were positively associated with HIV testing. Respondents who were aware of HCT services were more than four times more likely to get HIV testing in comparison with those without this knowledge (OR=4.15; 95% CI: 2.96–5.81). Individuals who cited stigma and discrimination as a social barrier to HIV testing were 56% less likely to test for HIV compared to those who indicated otherwise. Survey participants older than 25 years of age were 1.56 times more likely to seek HCT services compared to their younger counterparts (OR=1.56; 95% CI: 1.12–2.06).

**Table 3:** Multivariate analysis of factors associated with HCT by gender

	Overall			Female			Male		
			
Factor	Adjusted OR	95% CI	*P*-value	Adjusted OR	95% CI	*P*-value	Adjusted OR	95% CI	*P*-value
Gender									
Female	1 (Ref)								
Male	0.51	0.36–0.73	<0.001	–	–	–	–	–	–
Age group									
Less than 25	1 (Ref)			1 (Ref)			1 (Ref)		
More than 25	1.72	1.19–2.48	0.004	1.61	0.64–2.11	0.623	1.48	0.91–2.41	0.115
Marital status									
Single	1 (Ref)			1 (Ref)			1 (Ref)		
Cohabiting or married	1.31	0.88–1.94	0.178	0.83	0.43–1.59	0.569	1.68	1.02–2.78	0.041
Divorced	0.61	0.22–1.69	0.339	0.43	0.10–1.77	0.240	0.65	0.14–3.03	0.592
Education level									
Primary	1 (Ref)			1 (Ref)			1 (Ref)		
Secondary	1.48	0.99–2.17	0.047	0.66	0.32–1.35	0.255	2.00	1.15–3.47	0.014
Tertiary	1.58	1.05–2.52	0.033	0.77	0.32–1.82	0.549	1.99	1.11–3.29	0.020
Sexual partnerships									
None	1 (Ref)			1 (Ref)			1 (Ref)		
One	2.06	1.26–3.55	0.005	2.65	1.19–5.90	0.017	2.65	1.21–5.83	0.015
More than one	1.70	1.01–3.01	0.054	1.24	0.44–3.48	0.684	2.25	1.01–5.00	0.046
Distance to nearest clinic									
Less than 20 km	1 (Ref)			1 (Ref)			1 (Ref)		
More than 20 km	0.33	0.20–0.45	<0.001	0.21	0.12–0.39	<0.001	0.36	0.20–0.67	0.001
HCT awareness									
No	1 (Ref)			1 (Ref)			1 (Ref)		
Yes	4.01	2.77–5.81	0.000	3.42	1.72–6.81	0.000	3.76	2.32–6.10	0.000
Perceived benefit for HIV testing									
High	1 (Ref)			1 (Ref)			1 (Ref)		
Low	0.78	0.55–1.09	0.143	0.54	0.30–0.96	0.035	0.89	0.55–1.43	0.627
Stigma and discrimination as a social barrier to HIV testing									
No	1 (Ref)			1 (Ref)			1 (Ref)		
Yes	0.44	0.31–0.62	<0.001	0.59	0.33–1.04	0.068	0.35	0.22–0.54	<0.001

Multivariate analyses revealed that married men or those cohabiting with a partner were 1.68 (aOR=1.68; 95% CI=1.02–2.78) times more likely to test for HIV compared to single men. No association was observed in women. The older participants were more likely to seek HCT services compared to the younger ones (aOR=1.72; 95% CI=1.19–2.46), although no associations were observed between gender. In general, those with a tertiary education (aOR=1.63; 95% CI=1.05–2.52) were more likely to test for HIV compared to those with a primary school education. Men with a secondary (aOR=2.00; 95% CI=1.15–3.47) and tertiary (aOR=1.99; 95% CI=1.11–3.29) level of education were twice as likely to test for HIV. However, no such association was observed among women. Overall, participants who had one or more than one sexual partner were more likely to test for HIV compared to those who had none. In women, those with one sexual partner were 2.65 times likely to test for HIV (aOR=2.65; 95% CI=1.19–5.90). Men on the other hand, were likely to test for HIV whether they had one (aOR=2.65; 95% CI=1.21–5.83) or more than one sexual partners (aOR=2.25; 95% CI=1.01–5.00). Generally, those who lived further away from the nearest health centre were significantly less likely to undergo HIV testing. This trend was observed across gender. Participants who were aware of HCT were approximately four times more likely to test for HIV compared to those unaware of these services. Low perception (aOR=0.54; 95% CI=0.30–0.96) of the benefits for HIV testing reduced the likelihood of HIV testing in women only. This association was neither observed in men nor generally. Men who reported stigma and discrimination as a social barrier to HIV testing were less likely to test (aOR=0.35: 95% CI=0.22–0.54). This effect was observed overall but not in women.

## Discussion

The study revealed that, overall, HCT knowledge, having one sexual partner, being older than 25 years of age, living further away from a health facility, possessing a post-primary education, and having a low perception of the benefits of HIV testing were associated with HIV testing. Gender differences were identified: in women, having one sexual partner and HCT knowledge positively increased the chances for HIV testing, while living further away from health facilities and having a low perception of the benefits for HIV testing were negatively associated with HIV testing. In males, HIV testing was positively associated with being married or living with a partner, possessing post-primary school education, having one or more sexual partners, and HCT awareness. Similar to women, men who lived further away from clinics were less likely to undergo HIV testing.

Creating awareness of HCT services in the communities leads to an increase in the uptake of HCT. In this study, we found that awareness of HCT services improved the likelihood of testing as a high proportion of the participants in the survey who tested for HIV knew about HCT. Our findings are consistent with what was previously reported in Tanzania, where prior knowledge of HCT influenced the utilisation of HCT (34). Older individuals were more likely to use HCT services as opposed to the younger ones, and a possible interpretation would be that older people might perceive their HIV risk as high and would therefore readily access HCT services (35). According to our findings, men in sexual relationships were better targets for HCT uptake than single men. This finding is supported by a study conducted in rural western Uganda (15). Unlike women, men with post-primary education in this study responded better to calls for HIV testing than those below this educational stratum. Further research is warranted to better understand this phenomenon. A study conducted in the United States found that the percentage of men who tested for HIV did not vary significantly by education (36), a finding contrary to what was revealed in our study.

It is important to emphasise the benefits for HIV testing. We found that low perception of the benefits for HIV testing reduced the likelihood of HIV testing in women. No association was found in men or overall. Increasing knowledge on the benefits for HIV testing has been synonymous with an increase in the uptake of HCT in studies looking at antenatal care (37) and tuberculosis treatment (38). Our study identified stigma and discrimination as a social barrier affecting HIV testing. This low uptake of HIV testing in many settings of SSA has been associated with a heterosexual transmission synonymous with sexual promiscuity (6). Additionally, the stigma and discrimination association was observed in male rather than female respondents. This finding is in agreement with studies across SSA which revealed AIDS-related stigma and discrimination as major factors affecting the utilisation of HCT services (21). These studies further show that stigma reduces the efforts for a change in behaviour not only on an individual level but also in the community at large (2, 4, 6, 27, 30, 39).

## Limitations of the study

Although this study builds on the existing literature on HIV testing, several limitations were encountered which should be considered when interpreting the findings. One limitation was the cross-sectional nature of the survey data, making conclusions on causality impossible. Self-reported information in public venues would lead to over-reporting or under-reporting of socially desirable and unacceptable behaviours, respectively, affecting the accuracy of the data. It should also be noted that this study was conducted in one taxi rank out of many in the country, and therefore the results may not be generalised to the entire commuter population. Another potential limitation would result from non-representation of the population that use other forms of public transportation, such as trains and buses, which would result in sampling bias. The adjusted age group variable showed residual confounding which was identified during analysis by an increasing odds ratio. This may be attributed to possibly imprecise data on the confounding variables.

## Conclusion

There was low perceived benefit of testing for HIV that correlated with reduced odds of having been tested among women. Therefore, interventions to improve knowledge of HCT among women should be encouraged. More locations of appropriate testing centres or the introduction of home-based HCT would be required to increase HIV testing and counselling services. Continually addressing the stigma associated with HIV is important for ensuring high HIV testing rates.
